# Changes in sexual function and vaginal topography using transperineal ultrasound after vaginal laser treatment for women with stress urinary incontinence

**DOI:** 10.1038/s41598-022-06601-0

**Published:** 2022-03-02

**Authors:** Cheng-Yu Long, Pei-Chi Wu, Hung-Sheng Chen, Kun-Ling Lin, Zixi Loo, Yiyin Liu, Chin-Hu Wu

**Affiliations:** 1 Department of Obstetrics and Gynecology, Kaohsiung Medical University Hospital, Kaohsiung Medical University, No. 100, Ziyou 1st Rd., Sanmin Dist., Kaohsiung City, 807 Taiwan; 2 Department of Obstetrics and Gynecology, Kaohsiung Municipal Siaogang Hospital, Kaohsiung Medical University, Kaohsiung, Taiwan; 3 Regenerative Center, Kaohsiung Medical University, Kaohsiung, Taiwan; 4 Department of Obstetrics and Gynecology, National Taiwan University Hospital, Taipei, Taiwan; 5 Department of Obstetrics and Gynecology, Kaohsiung Municipal Da-Ton Hospital, Kaohsiung Medical University, Kaohsiung, Taiwan

**Keywords:** Diagnosis, Medical imaging, Ultrasonography, Outcomes research

## Abstract

We aim to assess the changes in sexual function and vaginal topography using 3-D transperineal ultrasound in stress-incontinent women treated with Er:YAG vaginal laser. Two hundred and twenty women with stress urinary incontinence (SUI) treated with Er:YAG laser were recruited. Assessment before and 6 months after the treatment included vaginal topography using 3-D transperineal ultrasound and sexual function using female sexual function index questionnaire (FSFI). A total of 50 women with complete data showed that the symptomatic improvement was noted in 37 (74%) women. After Er:YAG vaginal laser treatment, significantly decreased width and cross-sectional area in proximal, middle, and distal vagina were found in women with SUI. Nearly all of the domains of FSFI improved significantly after the vaginal laser treatment, except sexual desire. In conclusion, 3-D transperineal ultrasound can be used to conduct vaginal topography. After Er:YAG vaginal laser treatment, the anatomical changes of vaginal shrinkage and the improvement of female sexual function were both noted. The favorable outcome of sexual function partly related to the tightening of vagina, as evidenced by the measurements of the 3-D transperineal ultrasound.

## Introduction

The use of vaginal laser in urogynecology, such as genitourinary syndrome (GSM) and stress urinary incontinence (SUI) has become popular gradually in recent decade^1^ and shown potential as an alternative treatment to existing treatment options, such as topical hormonal cream, pelvic floor exercises or surgical procedures^2^. The non-ablative 2940 nm Er:YAG laser, using an erbium yttrium–aluminum-garnet medium with sequentially packaged bursts of long pulses of SMOOTH mode, is one of the most used vaginal laser for management of SUI^1,3^.

SUI was defined as involuntary loss of urine on effort or physical exertion including sporting activities, or on sneezing or coughing^4^, which was one of the most complained symptoms by the women visiting an urogynecological clinic. On the contrary, sexual dysfunction was rarely be the chief complaint. Literatures illustrating the improvement of sexual function after vaginal laser therapy for GSM are abundant. But most of the evaluation was assessed subjectively by questionnaire, and only one study assessed sexual function after treatment for SUI^1,5^, in which sexual function improvement after vaginal laser treatment for SUI was noted.

3-Dimensional (3D) Transperineal ultrasound is an easily approachable technique evaluating the pelvic anatomy with good reproducibility, and providing real-time data in urogynecological field^6^. It has been widely applied in pelvic organ prolapse (POP), SUI, defecatory dysfunction, structural defect and trauma, but no report assessing sexual dysfunction by 3-D transperineal ultrasound. For sexual function related image assessment, MRI was ever applied in the measurement of vaginal canal and revealed vaginal shrinkage after laser treatment^7^. Compared to MRI, 3D transperineal ultrasound is more cost-effective for anatomical evaluation.

We hypothesis that the 3-D transperineal ultrasound can be used to conduct a vaginal topography, which may correlate the changes of sexual function. We aim to assess the changes in sexual function and vaginal topography in SUI women treated with Er:YAG vaginal laser.

## Results

A total of 50 women were included in the final analysis in this study. The demographic data were shown in Table 1. Among those stress incontinent and sexually active women, the mean age was 47.1 ± 8.1 years, and the mean parity was 2.0 ± 0.6. Nineteen (38%) women were menopausal, and 5 (25%) women received hysterectomy before our recruitment of this study. Most of the women (86%) were mild to moderate SUI, and only 14% of them had severe symptoms of SUI. Improvement of SUI was found in 37 (74%) women.

**Table 1:** Demographic characteristics of the women received Er:YAG vaginal laser treatment for stress urinary incontinence (n = 50). Data are given as the mean ± standard deviation or n (%).

Parameters	Mean ± SD n (%)
Age (years)	47.1 ± 8.1
Parity	2.0 ± 0.6
BMI (kg/m^2^)	22.9 ± 10.8
Menopause	19 (38)
Hystery of hysterectomy	5 (25)
Diabetes mellitus	2 (4)
Hypertension	3 (6)
Severity of SUI*	
Grade I–II	43 (86)
Grade III	7 (14)

*BMI* body mass index, *SUI* stress urinary incontinence.

*By Ingelman–Sundberg method of stress incontinence classification. Grade I, mild: urinary incontinence when coughing or sneezing. Grade II, moderate: urinary incontinence when running or lifting objects off the floor. Grade III, severe: urinary incontinence when walking or climbing stairs.

In the objective assessment of vaginal topography via 3-D transperineal ultrasound, the cross-sectional area and width of vagina were both increased gradually from introitus to apex, no matter before or after vaginal Er:YAG laser treatment. The decrease in vaginal width and area after laser treatment were found not only in distal vagina, but also in middle and proximal vaginal, and the changes were all statistically significant (Table 2).

**Table 2:** Changes in vaginal topography using transperineal ultrasound before and 6 months after Er:YAG vaginal laser treatment for stress urinary incontinence (N = 50). Data are given as the mean ± standard deviation.

Parameters	Pre-treatment	6 months post-treatment	*p* value*
**Cross-sectional area (cm**^**2**^**)**			
Proximal vagina	2.4 ± 1.1	1.8 ± 0.8	< 0.001*
Middle vagina	2.2 ± 1.0	1.6 ± 0.8	< 0.001*
Distal vagina	1.4 ± 0.7	1.0 ± 0.5	< 0.001*
**Width (mm)**			
Proximal vagina	38.1 ± 8.3	34.2 ± 7.1	< 0.001*
Middle vagina	33.5 ± 6.2	29.0 ± 6.8	< 0.001*
Distal vagina	28.5 ± 9.2	23.5 ± 5.7	< 0.001*

*Paired t-test or Wilcoxon signed-rank test.

In the questionnaires assessing female sexual function, nearly all of the domains and total scores of FSFI were improved significantly after vaginal Er:YAG laser treatment for SUI, except sexual desire domain (2.8 ± 1.2 to 3.0 ± 1.0, *p* = 0.07) (Table 3).

**Table 3:** Changes in female sexual function index questionnaire (FSFI) before and 6 months after Er:YAG vaginal laser treatment for stress urinary incontinence (N = 50). Data are given as the mean ± standard deviation.

Domain of FSFI	Pre-treatment	6 months post-treatment	*p* value*
Desire	2.8 ± 1.2	3.0 ± 1.0	0.07
Arousal	3.0 ± 1.2	3.6 ± 0.9	< 0.001
Lubrication	4.1 ± 1.3	4.7 ± 1.0	< 0.001
Orgasm	3.8 ± 1.5	4.2 ± 1.3	0.024
Satisfaction	3.8 ± 1.7	4.5 ± 1.3	< 0.001
Pain	4.4 ± 1.3	5.2 ± 0.9	< 0.001
Total scores	22.2 ± 6.2	25.6 ± 4.5	< 0.001

*Paired t-test or Wilcoxon signed-rank test.

## Discussion

The thermal effect of non-ablative Er:YAG on human soft tissue lead to deep collagen remodeling and new collagen synthesis^8–10^. Collagen is the most important content of the endopelvic fascia, in which more than 3 quarters of protein is collagen^9^. After repetitive treatment with Er:YAG laser, the shortening of collagen fiber would cause tissue shrinkage and tissue retraction, followed by new collagen fiber formation^11–13^. The treated tissue would be enriched with new collagen and become tighter and more elastic^5^. Our study provide the image evidence of anatomical change in vaginal shrinkage after vaginal laser.

Following wide spread of concerns over the use of mesh in pelvic organ prolapse and SUI, alternative treatments were under investigation. In the last decade, the ability of collagen remodeling has been applied in several aspects of pelvic floor dysfunction. The injury of fascial hammock during childbirth and aging related poor collagen synthesis both lead to SUI^1^. Previous studies showed obviously diminished content of collagen in pubocervical fascia of incontinent women, both a quantitative and qualitative reduction^14,15^. In the latest review by C Phillips and colleagues^1^, there have been 15 publications assessing the efficacy of Er:YAG laser in SUI, and overall outcomes suggest an improvement at short-term follow-up. We provide vaginal laser as one of the optional treatment for SUI based on a shared decision-making principle in our daily practice^16^.

The female sexual dysfunction was rarely the chief complaint because of our implicit culture, but it is very common. Our study revealed that women with SUI, both premenopausal and postmenopausal, would have sexual dysfunction in every domain in FSFI. Some of the domains are related to condition of vaginal epithelium and vaginal laxity. Unlike SUI, vaginal laxity or vaginal relaxation syndrome is still not recognized as a medical condition, and the relationship between vaginal laxity and female sexual dysfunction was rarely studied.

In our study, significant improvements were found in most of the domains and total score in FSFI after treatment, except sexual desire. Low sexual desire is multifactorial, such as psychosocial and hormonal factors^17^. Thus, simply increasing the collagen synthesis is insufficient to improve sexual desire significantly, although we can still see an upgoing trend in the scores in desire domain, probably due to a better sexual experience after treatment. The most obvious improvements in FSFI were in lubrication, satisfaction and pain domains. These findings may be related to a better vaginal health in histological aspect, such as increased mucosal thickness, fibroblast activation, angiogenesis and restructuring^18^. Also, the improvement of SUI and GSM would reduce some embarrassing scenes, such as coital incontinence, dyspareunia and difficult penetration. These improvement would contribute to a better sexual experience and improve the desire and arousal of sex, resulting a better quality of life for these sexually active women with SUI.

Our study found significantly decreased vaginal width and cross-sectional area in proximal, middle and distal vagina in vaginal topography, using 3-D transperineal ultrasound. The thermal effect of Er:YAG laser could cause vaginal shrinkage, demonstrating the anatomical change in our result. Tien YW and colleagues had illustrated that Er:YAG vaginal laser improved both female and male sexual function^5^. An objectively smaller vaginal diameter may contribute to the tightening effect of vagina, and there might be a positive relation between decreased vaginal diameter, area, and better sexual function. Because the satisfaction of sex relates to the bidirectional interaction between partners^17^, a better vaginal mucosal condition and a tighter vagina can improve the partner response by positive male-to-female feedback. Although the improvement of sexual function was significant in our study, the total score after treatment still did not reach the suggested cut-off value of 26.55 in previous study^19^, indicating that a multidisciplinary treatment should be provided for female sexual dysfunction.

To the best of our knowledge, this is the first study using 3-D transperineal ultrasound to conduct vaginal topography and investigate the change of female sexual function after vaginal laser at the same time. The inter-observer and intra-observer reproducibility of transperineal ultrasound are good in different clinical situation^20,21^. The correlation with other image examination regarding vaginal topography, such as MRI, need further study. Previous study using pelvic examination and POP-Q for vaginal topography revealed no correlation between vaginal topography and sexual function^22^. However, pelvic examination cannot reach a precise measurement as ultrasound. Also, it is impossible to measure the proximal, middle, and distal cross-sectional area in pelvic examination.

The retrospective design of this study is the major limitation. Additionally, the follow-up period is short as 6 months. Although the follow-up period in most studies and ours were short, conservative treatment should be considered as a maintenance therapy, not curative, thus a short-term treatment effect evaluation is reasonable. The synthesis of collagen is not an once for all process. The fade-out of treatment effects and recurrence of symptoms are predictable in non-surgical treatment, and further study should work on the adequate vaginal laser treatment dosage and interval to achieve a better treatment plateau. Also, further study regarding questionnaire for male partners should be conduct to confirm that the anatomical changes in vaginal width and cross-sectional area are related to the sensation of tightness. Since this is the first study using 3-D transperineal ultrasound to conduct vaginal topography, the reliability, validity and correlation to MRI are still unknown and need further study. We conduct the protocol with the same operators, and this setting would provide more reliable data. This study provides new and useful information for pretreatment counseling in vaginal laser.

## Patients and methods

Between August 2015 and December 2017, a total of 220 women with SUI receiving Er:YAG vaginal laser treatment in the urogynecological department of a tertiary referral center were recruited. A retrospective chart review was performed. This study received approval from the Institutional Review Board of Kaohsiung Medical University Hospital (ID:KMUHIRB-E(I)-20180109), by which relevant guidelines and regulations were followed accordingly. The treatment principle, protocol, indication, and side effect of Er:YAG vaginal laser were well explained to the patient, and the decision of treatment choice were made based on the principle of shared decision-making with informed consent. Women who received additional treatment other than Er:YAG vaginal laser, with more or equal to stage 2 POP defined by POP-quantification system (POP-Q)^23^, with mixed urinary incontinence, non-sexually active, had chronic vaginal infection or inflammation, and with incomplete data for 6 months follow-up were excluded. Ultimately, the final analysis was conducted on the basis of 50 available subjects with complete follow-up.

The baseline demographic data included age, parity, body mass index, past medical history, previous surgical history and severity of SUI. “Sexually active” was defined as having vaginal intercourse at least 1 time per month within 6 months before treatment and continuously after treatment. The severity of SUI was classified into 3 grades based Ingelman-Sundberg method of stress incontinence classification^16,24^. Grade I, mild: urinary incontinence when coughing or sneezing. Grade II, moderate: urinary incontinence when running or lifting objects off the floor. Grade III, severe: urinary incontinence when walking or climbing stairs.

The Er:YAG vaginal laser (Fotona Smooth™ XS, Fotona, Ljubljana, Slovenia) with a wavelength of 2940 nm, was applied under original setting of instrument by the manufacture. The patient was set in lithotomy position without anesthesia. The vagina was irrigated with normal saline and dried with gauze. The laser delivery handpiece was inserted into the vaginal canal. The treatment protocol consisted of three phases. In the first phase, the probe rotated circularly, from the proximal part of vagina to the introitus, and the vagina was irradiated with a full spot R11 handpiece, with a fluence of 10 J/cm^2^ and 1.6 Hz in smooth mode. In the second phase, a fractional laser beam was emitted perpendicularly to the anterior wall using a PS03 handpiece, with fluence of 10 J/cm^2^ and 1.6 Hz in smooth mode for 5 passes. In the third phase, the introitus and vestibule were irradiated with a shooting pattern using a PS03 handpiece for 3 passes. The women received 3 treatment sessions with a 4-week interval.

**Figure 1:**
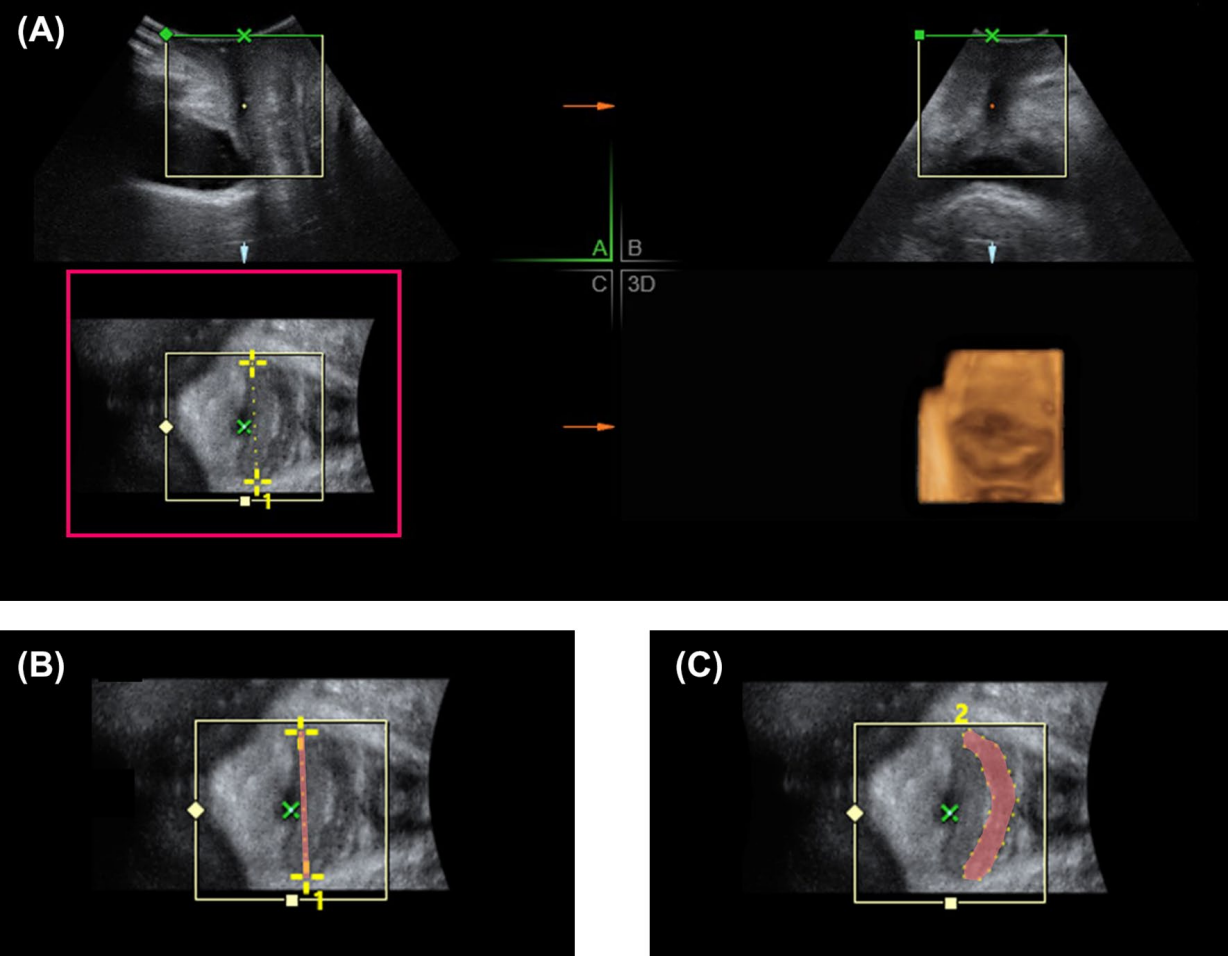
The measurement of vaginal topography via transperineal ultrasound. A convex probe was placed over perineum. The measurement was done on the midsagittal plane. Mode of rendered volume was obtained on relax without pelvic muscle contraction to obtained an axial plane of pelvis over the panel (**C**) in (**A**). The vagina was recognized as hypoechoic area between urethra and anal canal in genital hiatus. Vaginal width (the red line in **B**) and cross-sectional area of vagina (the red-marked area in **C**) were measured at level of proximal, middle and distal part of vagina.

**Figure 2:**
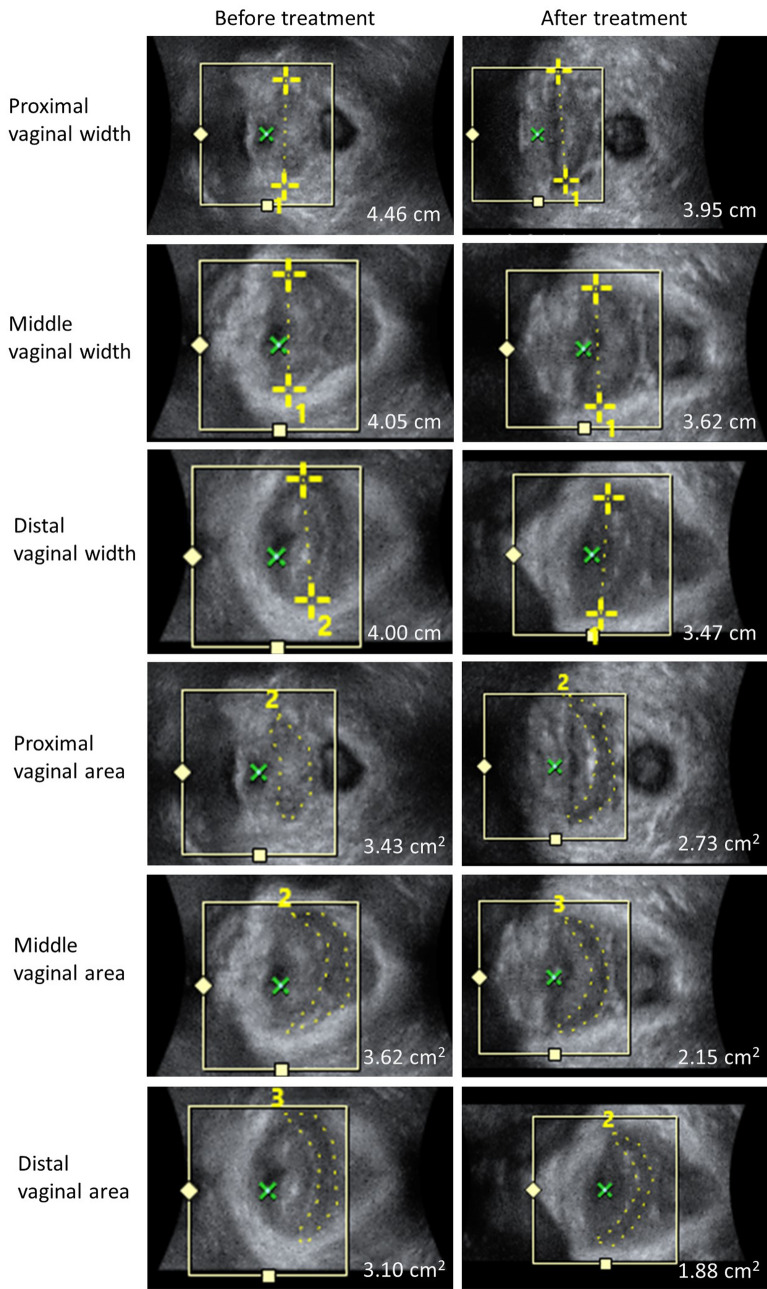
The sequential images of vaginal width and area of a same patient before and after treatment at different levels of vagina.

Before and 6 months after treatment, a 3-D transperineal ultrasound was applied for vaginal topography to those women with SUI received Er:YAG vaginal laser treatment. The ultrasound was performed by two experienced urogynecologist (CY Long and KL Lin) according to KMUH-TPUS protocol. In the protocol, the 3-D ultrasound was performed under a supine posture with bilateral hips and knees flexion and slightly spreading legs. A convex probe was placed over perineum. The measurement was done on the midsagittal plane. Mode of rendered volume was obtained on relax without pelvic muscle contraction to obtained an axial plane of pelvis over the panel C in Fig. 1A. The vagina was recognized as hypoechoic area between urethra and anal canal in genital hiatus. Vaginal width (the red line in Fig. 1B) and cross-sectional area of vagina (the red-marked area in Fig. 1C) were measured at level of proximal, middle and distal part of vagina. The middle vagina was defined as the vagina at bladder neck level. The proximal vagina was defined as 2 cm inside from the bladder neck, and the distal vagina was 2 cm outside from bladder neck. The sequential images of vaginal width and area of a same patient before and after treatment at different levels of vagina were shown in Fig. 2.

Each of the two ultrasound operators (CY Long and KL Lin), blinded to each other’s data, measured every parameter twice and the average of the 2 measurements was used for statistical analyses. Intra-observer reliability was evaluated using the measurements of the first investigator (CY Long), who performed two series of analysis, with an interval of 7–14 days between them, and was blinded to the previous analysis. Inter-observer reliability was assessed using the measurements of CY Long and KL Lin. A test–retest series for all data measured in all women showed good inter-observer agreement, and the intra-observer intraclass correlation coefficient ranged from 0.619 to 0.866.

The primary outcome of this study was the vaginal topography measurement of width and cross-sectional area in proximal, middle and distal vagina via 3-D transperineal ultrasound. The secondary outcomes were female sexual function assessment by Chinese version of female sexual function index (FSFI) questionnaire^25^.

### Statistics

IBM SPSS Statistical Software version 20.0 ed. was used for the statistical analyses. Paired *t*-tests and Wilcoxon signed-rank tests were performed for two related units on a continuous outcome. A *p*-value of less than 0.05 was considered statistically significant.

### Ethical approval

The study was conducted according to the guidelines of the Declaration of Helsinki, and approved by the Institutional Review Board Kaohsiung Medical University Hospital (ID:KMUHIRB-E(I)-20180109) on April 24th, 2018.

### Informed consent

Informed consent was obtained from all participants before surgeries.

## Conclusions

3-D transperineal ultrasound can be used to conduct vaginal topography. After Er:YAG vaginal laser treatment, decreased width and cross-sectional area in proximal, middle, and distal vagina were found in women with SUI. In addition, the scores of nearly all domains in FSFI were improved after treatment, except sexual desire, indicating the improvement of female sexual function. It is worth emphasizing that Er:YAG vaginal laser created a favorable outcome of sexual function, this partly related to the tightening of vagina, as evidenced by the measurements of the 3-D transperineal ultrasound.

## Data Availability

The datasets analyzed during the current study are available from the corresponding author on reasonable request.
